# Comparison between TAPP & Lichtenstein techniques for inguinal hernia repair: A retrospective cohort study

**DOI:** 10.1016/j.amsu.2021.103054

**Published:** 2021-11-11

**Authors:** Junaid Sofi, Fozia Nazir, Irfan Kar, Kaif Qayum

**Affiliations:** a Sher-i-Kashmir Institute of Medical Sciences, Srinagar, Jammu & Kashmir, India; b Hereford County Hospital, Wye Valley NHS Trust, Hereford, UK

**Keywords:** TAPP, **Transabdominal Preperitoneal**, Inguinal hernia, Transabdominal preperitoneal procedure, TAPP, Lichtenstein, SKIMS, SKIMS Medical College

## Abstract

**Background:**

Worldwide, inguinal hernia repair is one of the commonest surgeries. The best treatment option to primary hernia has been investigated, but there still remains lack of evidence about the ideal approach. Therefore, this study aimed to compare the outcomes of inguinal hernia repair using transabdominal preperitoneal procedure (TAPP) & Lichtenstein techniques.

**Materials and methods:**

This was a retrospective cohort study, conducted at Department of General & Minimal Invasive Surgery, SKIMS Medical College, Bemina, Srinagar. For performing the analysis, we used SPSS. Continuous variables were expressed as mean and standard deviation, and the categorical ones were presented as frequencies and percentages.

**Results:**

A total of 60 patients were included (30 in each group). The mean age of the patients in both groups was around 54 years, and all patients were males. In unilateral cases the operating time was greater in the TAPP group than the Lichtenstein group (p < 0.001); however, in the bilateral cases, the operating time was significantly greater in the Lichtenstein than the TAPP group (p = 0.003). The pain scores, in unilateral cases, were significantly lower in the TAPP group than the Lichtenstein group (p < 0.001). The overall complication rate in the TAPP group was 6.7% while in the Lichtenstein group it was at 23.3%. In unilateral and bilateral cases, the patients significantly returned to work earlier in the TAPP group than those in the Lichtenstein group (p < 0.001).

**Conclusion:**

TAPP and Lichtenstein techniques are both safe and reliable techniques for inguinal hernia repair. However, TAPP repair showed lesser post-operative pain, earlier discharge from the hospital, earlier return to usual activities, better cosmetic outcomes, and less persisting pain. However, there was no significant difference in the complication rate and TAPP repair was more costly for the patient.

## Highlights

• In unilateral inguinal hernia, operating time was significantly greater in TAPP group.
• In bilateral hernia, operating time was significantly greater in Lichtenstein group.
• In all cases, pain was significantly lower with TAPP than Lichtenstein groups.
• The overall complication rate was not significant between the two groups.
• Occult hernias on the opposite side could be identified in the TAPP group.

## 1 Introduction

Globally, inguinal hernia repair is a very commonly performed surgery. The number of patients undergoing this procedure, per year, exceeds 20 million [1]. Inguinal hernias patients are mainly symptomatic, necessitating surgery; but even those who are asymptomatic have a 70% risk of needing surgery within a period of 5 years following watchful waiting [2].

The risk factors for inguinal hernia development can be divided into risk factors related to the patient, for example, age and sex [3,4], and external factors like physically challenging work [5,6]. Lateral (indirect) hernias are more common; however, medial (direct) hernias are associated with a greater risk of recurrence following repair [7,8]. Both medial and lateral hernias are often treated in the same way, despite the variations in the age, sex, and recurrence rates denoting different etiologies [9].

The ideal treatment approach to primary inguinal hernia has been investigated globally; however, there is still a lack of evidence regarding the most favorable approach for recurrent inguinal hernia repair. Moreover, their appropriate method of surgery for this condition is debatable [10].

The Lichtenstein technique has been commonly used, amongst various tension-free surgical procedures, to repair both recurrent and primary inguinal hernias [11]. This anterior open method has been commonly performed by many surgeons since it can be done in local anaesthesia [12].

Meanwhile, the transabdominal preperitoneal procedure (TAPP) is a technique to repair the hernia by an intraperitoneal approach. TAPP can be beneficial for treating bilateral hernia repair, large hernia defects, and recurrence following open repair A large mesh can be placed with this technique to cover the direct, indirect and femoral spaces [13].

Therefore, this study aimed to compare the outcomes of inguinal hernia repair using TAPP & Lichtenstein techniques as regards to the operating time, post-operative hospital stay, return to work, cost effectiveness, complications, scar size, and the detection of clinically insignificant (occult) hernia on the contralateral side in TAPP.

## 2 Methods

### 2.1 Study design

This was a retrospective, cohort study which followed the Strengthening the reporting of cohort studies in surgery (STROCSS) guidelines for its proper conduction [14]. The study protocol was registered in Research Registry (UIN: researchregistry7206) [15].

### 2.2 Participants & approval

The included patients were between 18 years and 75 years, of either sex, having hernia of the following types: unilateral or bilateral uncomplicated inguinal hernia, primary or recurrent inguinal hernia, or direct and indirect inguinal hernia. The exclusion criteria were obstructed/incarcerated hernia, prior laparoscopic hernia repair(s), massive scrotal hernias, prior groin irradiation, untreated bladder outlet obstruction (Grade 3 prostatomegaly/stricture urethra). The study was conducted at the Department of General & Minimal Invasive Surgery, SKIMS Medical College, Srinagar over a period of two years (2015 - 2017).

### 2.3 Techniques

Transabdominal Pre-peritoneal Approach (TAPP) was compared with Lichtenstein's technique of tension-free mesh groin hernia repair. Details about the operation techniques are provided in the appendix. All the operations were performed by a single surgeon, assisted by a single assistant, well experienced in the inguinal hernia repair by either of the techniques. Patients included in the study were admitted a day prior to surgery. After the admission, a detailed history was taken and a physical examination was performed. The performed investigations were: haemogram, renal function test, liver function test, chest radiograph, electrocardiogram, and ultrasonography of the pelvis (to exclude prostatomegaly in patients above 40 years of age). Further relevant investigations (as and when necessary) were advised by the attending surgeon. An intravenous antibiotic was administered half an hour preoperatively in all cases. The patients were assessed daily till discharge and reassessed on follow-up at 7 days postoperatively for any complications and pain scores. The patients were further followed up for one month; pain score, return to work, scar size, recurrence, and complications (if any) were recorded as per the proforma.

### 2.4 Outcomes

The outcomes were the operation time, pain scores (Visual Analogue Scale, VAS), and complications including wound hematoma formation, wound seroma formation, wound infection, groin pain, early recurrence, postoperative hospital stay, return to work and scar size.

### 2.5 Cohort groups

The selected patients were stratified into two groups: Group 1 which comprised 30 patients, who underwent the TAPP repair for inguinal hernia repair, and group 2 which comprised 30 patients, who underwent the Lichtenstein technique for inguinal hernia repair.

### 2.6 Statistical methods

The recorded data was compiled and entered in a spreadsheet on Microsoft Excel and the analysis was performed on SPSS (SPSS Inc., Chicago, Illinois, USA). Continuous variables were expressed as mean and standard deviation, while the categorical variables were presented as frequencies and percentages. Student's independent *t*-test was employed for comparing continuous variables. Chi-square test or Fisher's exact test, whichever appropriate, was applied for comparing the categorical variables. Statistically significant data was considered when the p-value was less than 0.05. All P-values were two tailed.

## 3 Results

### 3.1 Participants

A total of 60 patients were included in the analysis. Each group (TAPP and Lichtenstein's techniques) had 30 participants.

### 3.2 Baseline characteristics

The mean age of the patients in the TAPP technique group was 54.4 ± 13.95 years compared with 54.2 ± 11.24 years in the Lichtenstein group. In the TAPP group, the youngest patient was 28 years old while the oldest was 75 years old. In the Lichtenstein group, the youngest patient was 30 years old, while the oldest was 70 years old. Using Analysis of Variance (ANOVA) no statistically significant variation in the age of patients was observed among the groups (p = 0.951). All included participants in both groups were males. In both groups, the right side was more commonly involved than the left side (46.7% in the TAPP group, and 50% in the Lichtenstein group). The two groups also showed no statistically significant variation for the side involved (p = 0.929). Direct hernias were more common in both groups (66.7% in the TAPP group, and 53.3% in the Lichtenstein group). With respect to the type of inguinal hernia, the two groups were statistically insignificant (p = 0.108). (Table 1).

**Table 1:** The table shows the baseline characters of the patients. Data are presented as Mean ± SD or number and percentage.

Baseline Characters	TAPP	Lichtenstein
Age (Years)	54.4 ± 13.95	54.2 ± 11.24
Males	30 (100%)	30 (100%)
Right-sided involvement	14 (46.7%)	15 (50.0%)
Left-sided involvement	11 (36.7%)	11 (36.7%)
Bilateral involvement	5 (16.7%)	4 (13.3%)
Direct	20 (66.7%)	16 (53.3%)
Indirect	10 (33.3%)	10 (33.3%)
Pantaloon	0 (0.0%)	4 (13.3%)

### 3.3 Outcomes

#### 3.3.1 Operation time (minutes)

In unilateral cases the operating time was greater in the case of the TAPP group (mean 55.2min) compared with the Lichtenstein group (mean 40.8min); which was statistically significant (p < 0.001). On the other hand, in the bilateral cases, the operating time was significantly greater in the case of the Lichtenstein group (mean 75.7min) compared with the TAPP group (mean 65.2min), p = 0.003 (Table 2).

**Table 2:** The table shows the outcomes for both groups. Data are presented as Mean ± SD or number and percentage.

Outcomes	TAPP	Lichtenstein
Operation Time (minutes) (Unilateral cases)	55.2 ± 11.43	40.8 ± 9.01
Operation Time (minutes) (Bilateral cases)	65.2 ± 7.08	75.7 ± 3.31
Pain scores (VAS) Day 0 (Unilateral cases)	31.2 ± 6.05	41.5 ± 4.81
Pain scores (VAS) Day 1 (Unilateral cases)	20.9 ± 8.11	29.3 ± 4.43
Pain scores (VAS) Day 7 (Unilateral cases)	7.9 ± 4.58	15.6 ± 4.93
Pain scores (VAS) Day 0 (Bilateral cases)	36.4 ± 3.29	44.3 ± 2.98
Pain scores (VAS) Day 1 (Bilateral cases)	23.6 ± 3.51	35.0 ± 3.16
Pain scores (VAS) Day 7 (Bilateral cases)	10.8 ± 2.95	18.3 ± 2.63
Postoperative stay (Days) (Unilateral cases)	1.7 ± 0.801	2.1 ± 0.711
Postoperative stay (Days) (Bilateral cases)	1.4 ± 0.89	3.3 ± 1.25
Overall complications	2 (6.7%)	7 (23.3%)
Return to work (Days) (Unilateral cases)	12.5 ± 5.25	20.3 ± 5.08
Return to work (Days) (Bilateral cases)	15.8 ± 0.836	25.3 ± 3.096

#### 3.3.2 Pain scores (VAS)

In unilateral cases the pain scores on postoperative day 0, day 1 and day 7 were significantly lower in the TAPP group (mean 31.2, 20.9, and 7.9 respectively) compared with the Lichtenstein group (mean 41.5, 29.3, and 15.6 respectively), p < 0.001 for each day. Regarding the bilateral cases the pain scores on postoperative day 0, day 1 and day 7 were also significantly lower in the TAPP group (mean 36.4, 23.6, and 10.8 respectively) compared with the Lichtenstein group, on the same days (mean 44.3, 35.0, and 18.3 respectively), p = 0.008 on day 0, p = 0.002 on day 1, and p = 0.006 on day 7 (Table 2).

#### 3.3.3 Postoperative stay (Days)

In unilateral cases, it was significantly lower in the TAPP group (mean 1.7) compared with the Lichtenstein group (mean 2.1), p = 0.046. In bilateral cases, it was also significantly lower in the TAPP group (mean 1.4) compared with the Lichtenstein group (mean 3.3), p = 0.036 (Table 2).

#### 3.3.4 Complications

The overall complication rate in the TAPP group was 6.7% while in the Lichtenstein group it stood at 23.3%. The spectrum of complications was different in the two groups, with wound infections and seromas & urinary retention being more common in the Lichtenstein group. One patient in the TAPP group had lateral injury to an inferior epigastric artery. The overall complication rate was not statistically significant (p = 0.148) between the two groups. In one patient, TAPP had to be converted to Lichtenstein procedure due to dense adhesions at the operative site (Table 2).

#### 3.3.5 Return to work (Days)

In unilateral cases the patients significantly returned to work earlier in the TAPP group (mean 12.5 days) compared with the Lichtenstein group (mean 20.3), p < 0.001. In bilateral cases, the patients also significantly returned to work earlier in the TAPP group (mean 15.8 days) compared with the Lichtenstein group (mean 25.3), p < 0.001 (Table 2).

#### 3.3.6 Others

The TAPP repair was associated with minimal skin scars (∼0.5 cm x 2 & 1 cm x1) at the port sites, without no an inguinal region scar, while the Lichtenstein repair had a large scar of around 6–8 cm size in the groin. In the TAPP group, the initial diagnostic laparoscopy revealed clinically occult contralateral inguinal hernias in two patients (6.66%).

## 4 Discussion

Operating times of surgical techniques vary between surgeons and also differ considerably between the centers. It is important as the time taken to perform the surgery can have cost implications. In this study, we observed that in unilateral cases the operative time for TAPP repair was significantly greater than that of the Lichtenstein repair. Meanwhile, in the bilateral cases we found that the operating time for TAPP repair was less than that of the Lichtenstein repair on the two sides in the same sitting, one after the other. A previous meta-analysis found a significant increase of 15.20 min in the mean operating time for laparoscopic inguinal hernia repair [16]. Sun et al. recently reported that the Lichtenstein technique could decrease the operative time [10].

In this study, one patient (3.3%) in the TAPP group had to be converted into the open repair on a table due to dense adhesions at the operating site. McCormack K et al. reported that 2.7% of the laparoscopic operations were converted to an open procedure among the 3130 allocated laparoscopic repairs [17].

In this study, mean pain scores in the TAPP group were 31.2 on day 0, 20.9 on day 1, and 7.9 on day 7, while in the Lichtenstein group they were 41.5, 29.3, and 15.6 respectively for the unilateral cases. In the bilateral cases, we found a similar trend, but slightly higher pain scores in both groups. On the other hand, Leigh Neumayer et al. [18] found out that on the day of surgery, the VAS in the laparoscopic group was greater than the open group but the score difference decreased after two weeks.

The lower pain scores in the TAPP group lead to earlier discharge from the hospital and earlier return to work. The difference was more prominent in the bilateral group with a mean postoperative stay of 1.4 days in the TAPP group compared to 3.3 days in the Lichtenstein group. Patients reported returning to work earlier in the TAPP group. This can be clarified by the non-existence of an inguinal incision or dissection of muscle in the groin during laparoscopic repair, the tension-free repair, and the lower complication rate.

In this study, we observed three cases (10%) of urinary retention, two cases (6.6%) of seroma formation, one case (3.3%) of wound infection, and one case (3.3%) of persistent pain in the Lichtenstein group. Two of the three urinary retention cases had grade 2 prostatomegaly and were started on alpha-blockers afterward. The patient with seroma was managed conservatively while the one with wound infection was treated with oral antibiotics. Mesh was preserved in both cases. The one patient with persistent pain at one month was managed conservatively using oral analgesics. In the TAPP group, one (3.3%) case of urinary retention and one (3.3%) case of inferior epigastric artery injury were encountered. The inferior epigastric artery injury was managed promptly by cauterization on the table. No case of seroma, hematoma, wound infection, visceral injury, or persistent pain was encountered.

As compared with open Lichtenstein repair, TAPP hernia repair was more costly to the patient and the healthcare facility; as it requires a laparoscopic setup, fixation device, and larger size mesh. Operative time is longer in the unilateral group but lower in the bilateral group, lower postoperative hospital stay, and earlier return to work. Also, the cosmetic results were certainly better in the TAPP group. The small (5 mm) port site scars were very much less noticeable than the large 6–8 cm groin scars. No early recurrence was recorded in our study in both groups; however, this study's follow-up duration was short.

Aiolfi et al. showed some similarities and other differences to our results. They reported that TAPP significantly decreased early postoperative pain, return to work, hematoma, and wound infection compared to the Lichtenstein tension-free repair. However, seroma and hospital length of stay were similar between them [19].

It was suggested that open, TAPP and other repairs were comparable in the short term and that further assessment on the long-term run is needed. Also, the choice of the best treatment option should depend on the surgeon's expertise and each patient [20]. Unfortunately, this study had a short follow-up, so we are highly recommending future research of a larger sample size and longer follow-up period.

The strengths include the lack of significant differences between the baseline characters, so preventing bias from other variables. However, the limitations of the study include the small sample size, which also resulted in no females' inclusion in the study. The study was a single-center - single surgeon study, so not ideal. We should have distinguished between the patients in whom tackers were used and the rest and its effect on the post-operative pain. Some patients could have been discharged on the same day of the surgery but we don't have a day-care setup in place for the same. Exact cost-effectiveness could not be estimated as the patients had to procure the consumable surgical supplies for themselves from the open market which didn't allow exact cost comparison. Lastly, the follow up in our study was too early to obtain long-term conclusions about the recurrence and chronic pain.

To conclude, TAPP and Lichtenstein techniques are both safe and reliable means of inguinal hernia repair. TAPP repair was associated with earlier toleration of oral feeds, lesser post-operative pain, earlier discharge from the hospital, earlier return to usual activities, and less persisting pain. However, there was no significant difference in the complication rate between the two techniques but there were higher chances of serious complications in the TAPP technique. TAPP repair had better cosmetic outcomes but was more costly for the patient. Also, occult hernias on the contralateral side could be visualized and could potentially be treated in the same sitting in the TAPP. Finally, more research is encouraged.

## Provenance and peer review

Not commissioned, externally peer-reviewed.

## Ethical approval

Not applicable as a retrospective cohort study.

## Sources of funding for your research

Not applicable.

## Author contribution

Study Concept: JS, FN Data Collection: JS,FN,IK. Data Analysis: JS, FN, KQ Writing Paper: JS, FN, IK, KQ.

## Registration of research studies

Name of the registry: Research Registry
Unique Identifying number or registration ID: researchregistry7206
Hyperlink to your specific registration (must be publicly accessible and will be checked): https://www.researchregistry.com/browse-the-registry#home/registrationdetails/6158d5826a1e4a001f167a2c/

## Consent

Not applicable.

## Guarantor

Junaid Sofi.

## Declaration of competing interest

No conflicts of Interest.

## Appendix

### Operation Techniques Details

#### Transabdominal Pre-peritoneal Approach (TAPP)

Following induction of general anaesthesia, the supine patient was placed on the operating table with the arms to the side and in a 10–20° Trendelenburg position. This helped with the reduction of hernias and allowed the intestines to gravitate into the upper abdomen. The TV monitors were placed at the patient's feet and the surgeon and the assistant stood on either side.

##### Establishment of Pneumoperitoneum and Trocar Placement

A Verres needle was inserted through a small supra-umbilical incision and a pneumoperitoneum of 12 mmHg pressure was created; followed by placement of a midline 10 mm camera port. With the camera inserted, the abdominal wall was *trans*-illuminated to allow for mapping of vessels and safe insertion of two 5 mm ports in the avascular area, under direct vision. The lateral ports were placed in the mid-clavicular line in the region of the umbilicus, the exact placement depended upon whether the repair was to be unilateral or bilateral. After ports were established, diagnostic laparoscopy of the entire abdomen was done to rule out other pathology or contraindications for surgery. Evaluation of the pelvis followed. Hernial defects were identified and determined whether they are direct or indirect defects. The emphasis was laid on the visualization of any occult hernia on the contralateral side. The operating surgeon stood opposite the side of the hernia and operated using both hands, one for the umbilical trocar and the other for the trocar on his side of the table (i.e. the side opposite the hernia defect.

##### Peritoneal Incision

The pre-peritoneal space was entered by incising the peritoneum transversely from the region of the anterior superior iliac spine continuing horizontally, in the medial direction to the lateral umbilical ligament.

##### Dissection of the Peritoneal Flaps

Peritoneal flaps were raised. If bilateral inguinal hernias were present, the surgeon and assistant first approached one side in this manner and then switched sides to repair the contralateral hernia. This was done in three steps:

(1) Lateral dissection on the space of Bogros,
(2) Medial dissection over the space of Retzius, and
(3) Central dissection over the site of the hernia and its hernia sac.

The cord structures were then dissected free of their peritoneal attachments. In a direct hernia, the peritoneal sac was pulled back within the peritoneal cavity with gentle traction to separate the thin peritoneal layer from the equally thin layer of transversalis fascia anterior to it. In an indirect hernia, the peritoneal sac was retracted off of the cord structures and pulled back within the peritoneal cavity.

##### Tailoring, Insertion, and Placement & fixation of the Prosthetic Mesh

The mesh was introduced into the abdominal cavity through the 10 mm port, after rolling it as compactly as possible over a Maryland dissector. The “rolling and unrolling” technique was fundamental to expedite the manoeuvers. It was rolled from the bottom up along the inner face of the mesh. On introducing the mesh, the distal tip was pointed (medial end) toward the pubic symphysis so that when it passed through the trocar the mesh unrolled exposing the lateral end. With the other Maryland dissector, the lateral end was pointed towards the anterior superior iliac spine allowing the mesh to finish extending and settle in its place. Before fixing the mesh it was checked that its placement extended well beyond (at least 3 cm around) the hernia defect, that its bottom edge extends below Cooper's ligament, and that the lower peritoneal flap could be lifted without rolling it. When the mesh was satisfactorily placed, it was either tacked into place or no tackers were used at all; tackers were applied to the Cooper's ligament. Further tackers were placed into the muscle layers anteriorly but none into the ileo-pubic tract or inferior to this. A maximum of 3 tackers were used in a case. If the hernia was bilateral, the same procedure was performed on the contralateral side, and a second mesh was used.

##### Closing the Peritoneal Flap & closing of Incisions

The peritoneum was then reconstituted by suturing with V lock sutures or Stratifix or Prolene and the operation was completed by closing the external oblique at the 10 mm port sites and placing subcutaneous sutures to the skin.

#### Lichtenstein Technique

Under spinal anaesthesia/general anaesthesia, a suprainguinal incision, 1 cm above and parallel to the inguinal ligament, was given and the subcutaneous fat was then opened along the length of the incision, and careful haemostasis was achieved by ligating superficial pudendal and superficial epigastric vessels(if encountered). The Scarpa fascia was similarly opened along the length of the incision, down to the external oblique aponeurosis, and the external inguinal ring and the lower border of the inguinal ligament are visualized.

##### Division of external oblique aponeurosis and exposure of inguinal canal

The external oblique aponeurosis was then opened along the line of incision, starting from the external ring and extending laterally for up to 5 cm. The ilioinguinal nerve, lying underneath the aponeurosis, was preserved wherever possible during the procedure. The superior and inferior flaps of the external oblique aponeurosis were gently freed from the underlying contents of the inguinal canal and overturned and separated to expose the cremaster with the cord structures, the ilioinguinal and iliohypogastric nerves, the uppermost aponeurotic portion of the internal oblique muscle, and conjoined tendon, and the free lower border of the inguinal ligament Wide separation of the two flaps provided ample space for placement and fixation of mesh under vision while protecting the nerves.

##### Dissection of the spermatic cord

The spermatic cord, along with the cremaster muscle, was then lifted up and separated from the pubic bone for about 2 cm beyond the pubic tubercle creating space for extending the mesh well beyond the pubic tubercle. The cord was encircled in a tape/gauze for ease of handling.

##### Identification and management of the hernial sac and placement of the mesh

The hernia sac was dissected off the cord and the cremaster muscle was divided longitudinally, the sac was either inverted (if small), divided and ligated, or resected. The contents were reduced and high ligation of the neck of the sac was performed. A 7.5 × 15 cm piece of polypropylene mesh was used for a Lichtenstein hernioplasty. On the medial side, the sharp corners of the mesh were trimmed to conform to the patient's anatomy. The mesh was trimmed to fit the space, with a slit cut laterally to accommodate the spermatic cord following the dissection of the spermatic cord from the hernial sac. The mesh was laid with the medial edge 1–2 cm medial to the pubic tubercle. It was fixed inferiorly first starting at the medial end with 2-0 polypropylene suture. Interrupted sutures were used to fix the lower edge of the mesh to the free lower border of the inguinal ligament up to a point just lateral to the internal ring. Three or four interrupted sutures will be used to fix the mesh superiorly to the rectus sheath safeguarding the iliohypogastric nerve. The two tails were then be overlapped lateral to the deep ring and secured by the two or three interrupted sutures making sure that the cord is not constricted. In male patients, the testis was always gently pulled back down to their normal scrotal position after fixation of the mesh.

##### Closure

Haemostasis was ensured in the inguinal canal, which was then closed by suturing the two flaps of the external oblique aponeurosis with continuous sutures using absorbable suture material. Subcutaneous tissue was approximated with interrupted sutures to obliterate any dead space and the skin was approximated with interrupted 2-0 vicryl rapide or silk sutures or continuous subcuticular Prolene Nylon sutures and the operative site was cleaned and a sterile dressing applied and scrotal support given.
